# The role of predictive and preview effects in Mongolian reading: evidence from eye movements

**DOI:** 10.3389/fpsyg.2024.1420223

**Published:** 2024-09-13

**Authors:** Zhang Lu, Na Ri, Wang Jingxin

**Affiliations:** ^1^Faculty of Psychology, Tianjin Normal University, Tianjin, China; ^2^School of Education Science, Inner Mongolia Minzu University, Tongliao, Inner Mongolia, China; ^3^Research Base of Inner Mongolia Education and Psychological Development, Tongliao, Inner Mongolia, China

**Keywords:** Mongolian, boundary paradigm, predictive, preview, word skipping

## Abstract

**Introduction:**

The research on contextual predictability in reading has been thoroughly investigated in the context of horizontal text comprehension. However, the performance of contextual predictive effects in Mongolian vertical reading remains unknown.

**Methods:**

To explore this, we conducted an eye-tracking study using a boundary paradigm. Our study aimed to investigate contextual predictability and preview effects in Mongolian reading.

**Results:**

We found significant main effects of predictability and previewing on temporal indicators. However, there were no significant effects on skipping rates, and no interaction between predictability and previewing was observed.

**Discussion:**

We speculate that the unique reading orientation and writing features of Mongolian, compared to horizontally read phonetic scripts, reduce the parafoveal processing of preview information, leading to lower skipping rates in Mongolian reading.

## 1 Introduction

In natural reading contexts, vocabulary is typically embedded within sentences, its processing is intricately woven with contextual cues. The ability of readers to anticipate forthcoming words based on preceding context, known as contextual predictability (Rayner and Well, 1996), has garnered significant attention. Across diverse age groups (Choi et al., 2017; Liu et al., 2019; Rayner et al., 2006) and languages spanning English, German, and Chinese, high-predictability vocabulary tends to result in shorter fixation durations and a higher rate of skipping (Balota et al., 1985; Drieghe et al., 2005; Rayner et al., 2006, 2011; Rayner and Well, 1996; Staub, 2015). In eye movement control models such as E-Z Reader (Reichle et al., 1998) and SWIFT (Engbert et al., 2005), predictability plays a critical factor that finds robust emulation and application. These models effectively emulate the effects of high predictability on fixation durations and skipping rates.

It is evident that much of the research on predictability has revolved around horizontally read texts, primarily those in Latin scripts, such as English and German, read from left to right. However, a recent eye-tracking study on the predictability effects in Arabic (read from right to left) found that specific orthographic and morphological characteristics of the script itself can Decrease skipping rates (Aljassmi et al., 2022, Experiment 1). In the study, the predictability of words, ranging from four to eight letters and varying in length and morphological complexity, did not affect skipping probabilities (Experiment 1). However, predictability did influence skipping probabilities for shorter words (three to four letters) with simpler structures (Experiment 2). The increased word length and morphological complexity in Arabic heightened the processing demands on the parafoveal region, resulting in non-significant differences in skipping rates across varying levels of predictability.

Indeed, word skipping rates are influenced by both the predictability of the target word and the quality of parafoveal preview information. The influence of preview on predictability seems to partly determine whether readers can engage in efficient reading. Both contextual predictability and preview are crucial factors for ensuring smooth reading. During reading, readers not only anticipate forthcoming content through contextual cues but also begin processing words within the parafoveal span before fixating directly on them in the fovea. The phenomenon where information previewed in the parafovea facilitates the processing of the subsequent target word is known as the preview benefit (PB) (Rayner, 2009). This effect underscores the importance of parafoveal processing in enhancing overall reading efficiency. In reading, the ability to process information in the parafoveal region significantly contributes to improving readers’ overall reading performance. Research on parafoveal preview processing has shown that information at various levels can be extracted during the preview stage (Rayner, 1975; Schotter et al., 2012; Zhang et al., 2020; Zhu et al., 2021). The boundary paradigm stands out as one of the most widely employed methods to investigate preview processing (Rayner, 1975). In experiments, researchers commonly employ an invisible boundary preceding the target word within the sentence. The target word is presented under either a valid preview condition (where the preview word matches the target word) or an invalid preview condition (where the preview word is a pseudoword or a random string, etc.). Once the reader’s gaze crosses this invisible boundary, the target word is immediately replaced with the correct word. This process occurs swiftly, often without the reader’s awareness of the alteration.

In an early study, Balota et al. (1985) utilized the boundary paradigm to concurrently examine predictability and preview processing. Predictability was assessed through a cloze task, which measured the likelihood of a word filling in the blank. They designed the preview conditions to include five types, includes identical, semantically related, visually similar, visually dissimilar and anomalous. The results demonstrated that contextual predictability effects on gaze duration were evident under identical and visually similar parafoveal preview conditions, while these effects diminished under the other three conditions. The impact of contextual predictability on lexical processing depends on the preview content being identical to the target word. The predictability effect only emerges when the preview information matches the target word, resulting in shorter fixation times and more word skipping for highly predictable words. Under different preview conditions, the lack of matching preview information leads to the disappearance of the predictability effect (Staub and Goddard, 2019). It appears that the manifestation of early predictability effects during reading relies on the effectiveness of early bottom-up preview information. Similar findings have been confirmed in subsequent empirical studies on English (Staub and Goddard, 2019; Veldre and Andrews, 2018; White et al., 2005) and Chinese (Chang et al., 2020). This is reflected in the experimental results as a significant interaction between contextual predictability and preview. These studies converge on a conclusion that predictive processing occurs only when early parafoveal preview information is valid; however, when this preview information is invalid, predictive processing based on contextual cues weakens or fails to take place (Chang et al., 2020; Staub and Goddard, 2019).

However, it is undeniable that the invalid preview conditions (pseudowords, unrelated words) still contain lexical elements. This could potentially interfere with the processing of the target word and increase processing costs (Angele et al., 2016; Yang and Yu, 2021; Marx et al., 2015; Vasilev et al., 2018). The absence of predictability effects May not necessarily be due to a lack of effective preview but could also be attributed to interference caused by invalid preview (Parker et al., 2017; Parker and Slattery, 2019). According to Parker et al. (2017), if the invalid preview consists of unrelated words that conflict with the expected content, it can interfere with the target word, thereby affecting the manifestation of the contextual predictability effect. However, even in such cases, the contextual predictability effect May still be reflected in temporal measures (e.g., gaze duration), specifically by showing reduced fixation times and increased skipping rates. Zhao et al. (2023) used the incremental preview paradigm (Warrington et al., 2018) to investigate preview effects. This paradigm controls the presentation of stimuli to the right of fixation and includes conditions without preview to eliminate interference from irrelevant preview information. It was found that the interaction between contextual predictability and preview was not significant, as the contextual predictability effect was observed even in the absence of preview, similar to the effect seen with matching preview conditions. They directly tested the viewpoints regarding the lack of effective preview and interference from invalid preview in this controversy. Their findings supported the hypothesis that contextual predictability effects do not depend on having a preview identical to the target word, aligning with Parker et al.’s (2017) interference hypothesis.

This study aims to explore predictability and preview effects by introducing a Novel language paradigm. We delve into these phenomena further by employing Mongolian, a language read vertically, thus extending our investigation beyond existing research paradigms. Furthermore, it will provide valuable data references for eye movement control models. Given that previous research primarily focuses on horizontally written languages, there is a notable lack of exploration regarding vertically written Mongolian. Additionally, our use of vertically oriented text processing reflects the natural reading direction in Mongolian, rather than employing an unconventional vertical orientation which could lead to confusion (Yu et al., 2010).

A recent eye-tracking study conducted by Su et al. (2020) using Mongolian found that perceptual span exhibits “flexibility” in reading direction, whether horizontal or vertical. Borjigin et al. (2024) further investigated and found that the perceptual span in Mongolian reading is asymmetrical downward. Specifically, it extends one syllable upward and three syllables downward. In Mongolian writing, words are written as units with characters connected vertically, and the reading direction is from top to bottom. In addition to its distinctive reading direction, Mongolian is characterized by distinct orthographic features. Specifically, Mongolian words appear as continuous strings without structural boundaries between characters. Each character has three different forms to reflect its position within a word (initial, medial, and final).

Although the writing and reading characteristics of Mongolian differ from those of other widely studied languages, research shows that the perceptual span asymmetry observed during vertical Mongolian reading is consistent with the asymmetry found in horizontal writing texts (Borjigin et al., 2024). This consistency in asymmetry across reading directions reveals a universal feature of human visual information extraction. It can be said that the reading strategies and habits of Mongolian readers play a key role in adapting to vertical writing, as they have developed processing systems optimized for vertical reading. This adaptation May compensate for the visual processing limitations caused by horizontal-vertical anisotropy (HVA, Rovamo and Virsu, 1979). Consequently, Mongolian readers can achieve smooth reading in the vertical direction, and naturally begin pre-processing of words within the parafoveal region before directly fixating on the words in the fovea. However, whether the preview effects are similar to those observed in horizontal reading remains to be further investigated.

However, research on Mongolian has predominantly focused on orthographic processing and phonological activation (Hou and Qi, 2019) and studies on phonological automatic activation (Hou and Bai, 2013). Therefore, this study aims to enrich our understanding of eye movement characteristics in Mongolian reading by examining predictability and preview effects, and also provide a foundational basis for future in-depth exploration of reading mechanisms in Mongolian.

We employed the boundary paradigm (Rayner, 1975) to investigate the role of predictability and parafoveal preview effectiveness in Mongolian reading. Mongolian-derived words were used as target words in the experiments. Controlling for variations in predictability levels and preview effectiveness of target words, we investigated the impact of predictability on eye movement measures during reading by examining effects on fixation times and skip rates, and exploring the influence of parafoveal preview processing. Given the distinctive reading direction and orthographic features of Mongolian, similar predictability effects as those observed in Arabic script May occur. High predictability words might exhibit shorter fixation durations without affecting the skipping rate.

## 2 Methods

### 2.1 Participants

We recruited a random sample of 40 Mongolian undergraduate students aged 17–21 years (*M* = 18.45, *SD* = 0.64). All participants were native speakers of Mongolian with normal or corrected-to-normal vision, and received education in Mongolian upon enrollment. Their parents are proficient users of Mongolian as well. Upon completion of the experiment, participants received course credits as incentives. Written consent was obtained from all participants before the commencement of the study.

### 2.2 Stimuli and design

The experiment comprised 80 sets of sentences as reading materials, with each set containing four conditions as per the experimental design: high predictability – valid preview, high predictability – invalid preview, low predictability – valid preview, low predictability – invalid preview.

Before the formal experiment, 15 Mongolian undergraduate students were recruited to assess the predictability of the sentences. This was achieved using a classic cloze task, where participants were required to predict the target word itself (represented by parentheses) based on the preceding sentence context. The target word was assessed for contextual predictability based on the frequency with which it was predicted by participants. According to previous research, words with a cumulative probability of filling in the blanks exceeding 67% were classified as having high contextual predictability, while those below 17% were deemed to have low contextual predictability (Zhao et al., 2023).

Additionally, 20 Mongolian undergraduate students were recruited to rate the plausibility of the sentences on a 7-point scale (where “7” indicates very coherent and “1” indicates very incoherent). The experimental materials were matched for target word frequency, word familiarity, word length, and sentence plausibility based on these assessments (see Table 1). The sentences averaged 11 characters in length (ranging from 8 to 16 characters), and the target words consistently appeared near the middle of the sentences.

**Table 1:** Properties of target words.

Variables	Word predictability		Inferential statistics	
	High predictability	Low predictability	*t*	*p*
Word predictability	0.76	0.11	40	<0.001
Word frequency (per million)	591.66	534.69	0.27	0.78
Word familiarity	5.93	5.78	1.57	0.12
Word length	6.45	6.81	1.17	0.24
Sentence plausibility	6.02	5.99	0.38	0.70

The study employed a 2 (Predictability: High, Low) × 2 (Preview Type: Valid, Invalid) within-subject design. Under the valid preview condition, the preview word was identical to the target word, both being genuine Mongolian words. In the invalid preview condition, the preview word was an unrelated Mongolian pseudoword, with the same word length as the target word and ensuring that none of the letters in the pseudoword matched those in the target word.

The 80 sets of experimental materials were grouped, with each group containing an equal number of sentences for each condition. Furthermore, all 60 filler sentences generated were Declarative, unambiguous statements, with an average length matching that of experimental sentences. None of the target words were used in the filler sentences. Each participant read one set of experimental materials, with 25% of the experimental sentences followed by a yes/no comprehension question. Each participant read a total of 80 experimental sentences, 60 filler sentences, and 10 practice sentences, totaling 150 sentences. These practice sentences will not reappear in subsequent formal experiments.

We conducted a power analysis following the method outlined by Brysbaert and Stevens (2018). The results indicate that the power for our eye-tracking measures is 1.00, which significantly exceeds the commonly accepted threshold of 0.80. This confirms that our study’s design, including the number of participants and items, is sufficient to detect the interaction effects in our 2 × 2 within-subjects design.

**Figure 1:**
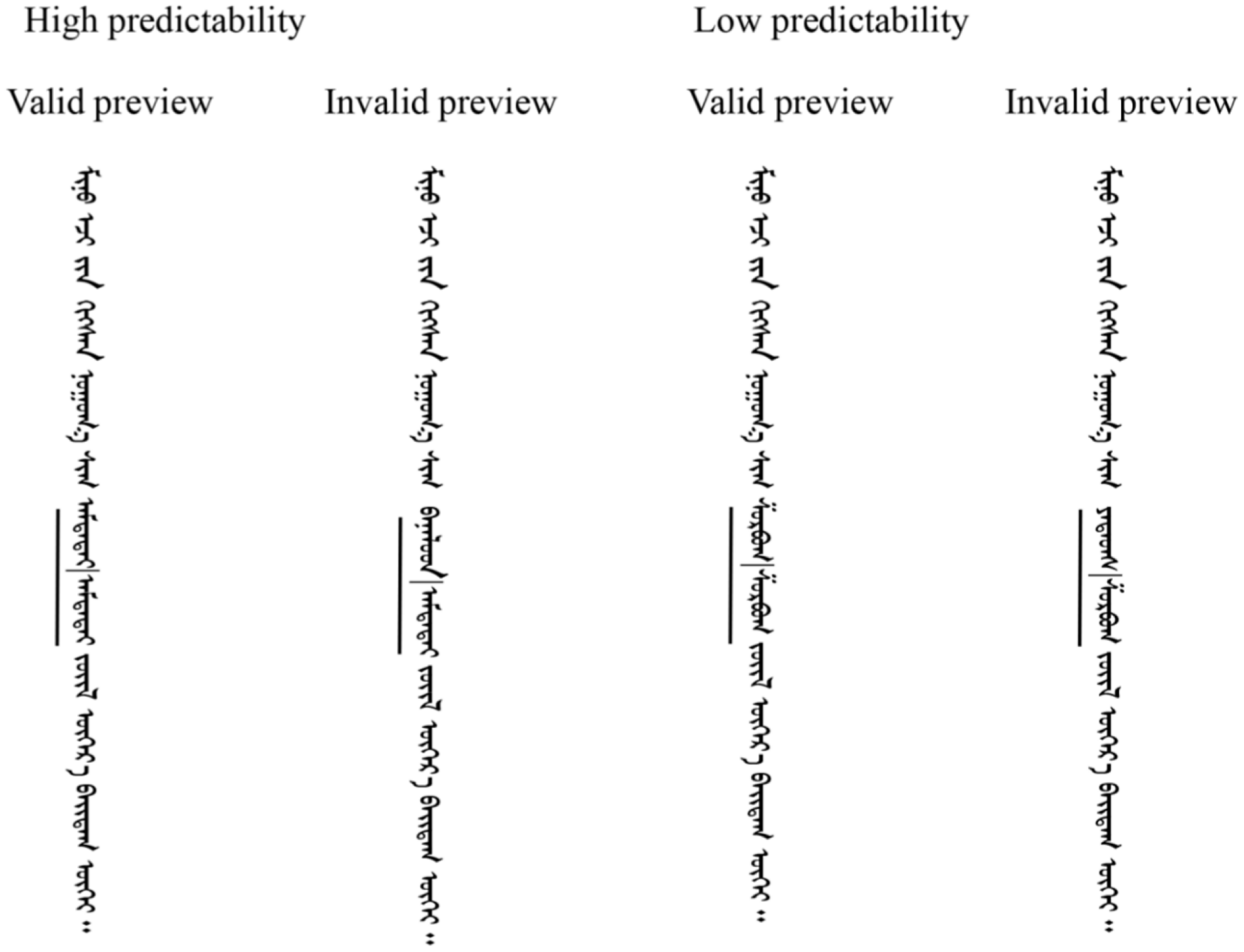
Example sentences in each condition. The high predictability sentences translated as “*There is nothing as delicious as the dishes my mother makes.”* The low predictability sentences translated as *“There is nothing as salty as the dishes my mother makes.”* The left side of the target word is underlined.

An example of the experimental materials is provided in Figure 1.

### 2.3 Apparatus and procedure

The present study utilized the EyeLink 1,000 Plus eye tracker manufactured by SR Research, Canada, to record participants’ eye movement trajectories during reading. The eye tracker operated at a sampling rate of 1,000 Hz. The Mongolian text in the sentence materials was presented in 28-point Menk Qagan Tig font. Sentences were rendered as images with black text on a white background and displayed on a monitor with a screen resolution of 1,024 × 768 pixels and a refresh rate of 60 Hz. Each letter subtended approximately 1° of visual angle.

During the formal experiment, each participant underwent testing individually in a laboratory room, maintaining a standard distance of 60 cm from the computer screen. Participants first familiarized themselves with the laboratory environment and adjusted to a comfortable sitting position before reading the instructions displayed on the computer screen. Subsequently, the experimenter explained the experimental procedure, requirements, and instructions, emphasizing the prohibition of reading aloud the sentences presented on the screen. Prior to commencing the experiment, three calibration steps were conducted to ensure accuracy, with both the maximum and average calibration errors being less than 0.3°. After completing practice sentences across 10 trials, participants proceeded to the formal experimental phase. This phase comprised 80 experimental sentences and 60 filler sentences, with identical filler sentences within each experimental group. Within each trial, a “+” symbol initially appeared at the position of the first letter of the sentence. Participants were required to accurately fixate on the “+” before the sentence appeared on the screen. Following reading, participants advanced to the next trial by pressing the space bar. After every third trial, a comprehension question (yes/no) was presented. Participants responded using the “F” key for “yes” and the “J” key for “no” on the keyboard.

The comprehension accuracy and responses were used to assess participants’ attentiveness to the reading task. The entire experiment was anticipated to last approximately 25 min.

## 3 Results

Data files and associated resources can be accessed through the University of Leicester’s Figshare repository online: https://doi.org/10.6084/m9.figshare.26405089.

The participants demonstrated a comprehension accuracy exceeding 80% in answering the questions. Data filtering procedures were conducted as follows according to standard protocols: fixations shorter than 80 ms and longer than 1,000 ms were removed. Additionally, data from trials with tracking losses (6 trials, 0.2%), trials with fewer than 5 fixations (18 trials, 0.6%), trials were excluded due to blinks occurred crossing the boundary or fixating on the target region (40 trials, 1.25%), and trials were also excluded if a display-change occurred prematurely before the reader’s gaze crossed the invisible boundary, or if it occurred late (161 trials, 5.03%).

Using R statistical software (R Development Core Team, 2016) with the lme4 package (Bates et al., 2015), linear mixed-effects models (LMM) were employed to analyze continuous variables. The results were reported with the fixed effects’ *b* values (i.e., coefficients, indicating the magnitude of fixed effects), standard errors (*SE*), and the corresponding *t*/*z* values and *p*-values for the tests. We used maximum random effects structures (Barr et al., 2013) for all measures. Fixed factors included word predictability, preview validity, and their interaction, with participants and stimuli treated as crossed random effects. In cases where the maximum random effects model did not converge, we simplified the random structure for stimuli, starting with the removal of random effect correlations, followed by random slopes reduction.

The dependent variables analyzed included: skip rate (SKIP, the proportion of words not fixated during the first reading), first fixation duration (FFD, the duration of the first fixation on the target word during the first pass reading), single fixation duration (SFD, the duration of the first fixation on the target word when it receives only one fixation during the first pass reading), gaze duration (GD, the total duration of fixations on the target word during the first reading), total reading time (TRT, the total duration of fixations on the target word) (Yan et al., 2013).

The eye-tracking data analysis revealed significant main effects of predictability and preview type on all reading times. Readers exhibited shorter reading times for high predictability words compared to low predictability words in Mongolian. Under valid preview conditions, reading times were shorter than under invalid preview conditions. However, there were no significant main effects of predictability and preview type on skip rate, and the interaction effects were non-significant for all measures (see Tables 2, 3).

**Table 2:** Means for target word measures.

Measures	High predictability		Low predictability		Preview effect	Predictability effect
	Valid preview	Invalid preview	Valid preview	Invalid preview		
SKIP (%)	12.37 (0.01)	9.73 (0.01)	10.41 (0.01)	11.07 (0.01)	0.99	0.31
FFD (ms)	274 (5)	309 (5)	295 (5)	326 (6)	33	19
SFD(ms)	286 (6)	327 (6)	310 (6)	345 (7)	38	21
GD (ms)	353 (8)	389 (7)	393 (8)	436 (9)	40	44
TRT(ms)	390 (11)	456 (11)	483 (13)	568 (14)	76	103

Standard deviations are provided in parentheses.

**Table 3:** Summary of statistical effects.

Measure	Effect	*b*	SE	*t*/*z*	*p*
SKIP	Word predictability	−0.03	0.12	−0.28	0.78
	Preview validity	−0.11	0.12	−0.87	0.38
	Word predictability × Preview validity	0.37	0.24	1.55	0.12
FFD	Word predictability	18.25	5.63	3.24	0.002
	Preview validity	33.69	7.46	4.51	<0.001
	Word predictability × Preview validity	−6.06	11.72	−0.52	0.61
SFD	Word predictability	20.68	6.57	3.15	0.002
	Preview validity	38.13	9.29	4.10	<0.001
	Word predictability × Preview validity	−8.77	13.08	−0.67	0.51
GD	Word predictability	43.12	10.00	4.31	<0.001
	Preview validity	40.32	10.52	3.83	<0.001
	Word predictability × Preview validity	2.71	15.65	0.17	0.86
TRT	Word predictability	103.51	19.35	5.35	<0.001
	Preview validity	74.67	14.66	5.10	<0.001
	Word predictability × Preview validity	16.08	24.37	0.66	0.51

To further assess the reliability of the finding that there is no interaction effect between predictability and preview on FFD, SFD,GD, and TRT, we conducted a Bayesian analysis using Bayes Factors (Morey et al., 2018). We utilized the Bayes Factor package to perform linear mixed model analyses for each of these eye-tracking metrics. Specifically, we compared a full model (*BF_Full_*), which included both the main effects and their interaction, to a model with only the main effects (*BF_Main_*). The Bayes Factor (*BF*) was computed as the ratio of *BF_Full_* to *BF_Main_*.

In our analysis, we employed a default prior probability of 0.5 and conducted 100,000 Monte Carlo iterations. The results indicated that all *BF* values were less than 1 (FFD, 1:13.46; SFD, 1:12.23; GD, 1:14.90; TRT, 1:12.92), providing strong evidence in favor of the null hypothesis and supporting the conclusion that there is no significant interaction effect between predictability and preview.

## 4 Discussion

Our findings represent a Novel contribution to the literature on prediction and preview effects. Through the implementation of a boundary paradigm eye-tracking experiment, we provide the first empirical evidence of context predictability and preview effects in vertical text reading, although we did not detect any interaction between the two. In contrast to prior research, our findings suggest a reduced tendency for skipping in Mongolian language reading across conditions of high/low predictability and valid/invalid preview. Particularly intriguing is the significant main effect of predictability and preview on temporal measures, while the absence of significant interaction effects across all temporal and saccadic measures suggests that predictability effects operate independently of the validity of previewing information. Our results indicate that Mongolian May be characterized as a language with fewer instances of skipping during reading. Below, we provide an interpretation of our findings.

We observed shorter fixation duration for target words under conditions of high predictability compared to low predictability in Mongolian text reading. However, unlike findings in studies on Latinate scripts such as English and German, where predictability significantly affects skipping rates, our research revealed a different pattern. Specifically, the average skipping rate for target words in our study was 10.90%, Markedly lower than observed skipping rates reported in eye-tracking studies on German (Kliegl et al., 2004) and Hebrew (Deutsch and Rayner, 1999) under similar word-length conditions. However, our findings are not entirely inconsistent with those observed in studies involving horizontally read scripts. As discussed in the introduction, Aljassmi et al. (2022) investigated predictability effects in Arabic, and similarly reported a low average skipping rate of 7.5% in their Experiment 1. The textual materials utilized in their study exhibit a right-to-left reading orientation and morphological complexity, characteristics that resonate with similar features found in Mongolian. Their research revealed pervasive predictability effects on fixations times (e.g., first fixation duration, total gaze duration) in Arabic (Experiments 1 and 2). However, due to specific orthographic and morphological features of the script, leading to poor parafoveal processing, the impact on skipping rate indicators was limited to shorter, morphologically simpler words (Experiment 2).

It seems plausible to suggest that the low skipping rate observed in Mongolian reading May also be closely related to the average word length (6.5 characters in our study). Word length is known to be one of the factors influencing skipping rates. The more characters within a word, the lower the skipping rate (German, Deutsch and Rayner, 1999; Hebrew, Kliegl et al., 2004). Mongolian words exhibit significant variation in character length, resulting in flexible word lengths where words containing the same number of characters May differ widely in spatial length. In languages where the number of characters within words does not correspond to spatial length, character count influences gaze duration, while spatial length affects skipping rates (Arabic, Hermena et al., 2017; Finnish, Hautala et al., 2011). In Mongolian, word length not only exhibits spatial flexibility but also operates along the vertical axis from top to bottom.

Different from the horizontal reading patterns of English (left to right) and Arabic (right to left), Mongolian adopts a vertical writing and reading style from top to bottom. It is widely acknowledged that language processing is predominantly associated with the left hemisphere of the brain (Jordan et al., 2014), making the left-to-right reading direction more aligned with human cognitive patterns. Compared to vertical reading, horizontal reading offers greater spatial freedom and more flexible eye movement control (Collewijn et al., 1988), thereby potentially enhancing language processing efficiency. Similarly, the larger perceptual span in the right visual field than in the left allows more letters to project to the left hemisphere. The asymmetry in sensitivity to visual and language information between the upper and lower visual fields May impair vertical reading (Goldstein and Babkoff, 2001; Hagenbeek and Van Strien, 2002; Yu et al., 2014). In studies examining horizontal reading of phonetic scripts, such as English, the collection of eye movement data focuses on the right of the fixation point, delineating the preview range. However, for Mongolian, which employs vertical reading, peripheral vision during reading presents information below the fixation point. Although Mongolian readers are capable of fluent vertical reading, their reading strategies and habits seem to ensure that information extraction at the parafoveal region remains unaffected. However, this does not necessarily prevent any impact on the overall information processing.

Our study reveals that the parafoveal processing effects observed in Mongolian contrast with the results reported in the meta-analysis by Vasilev et al. (2016). The complexity and difficulty of parafoveal information processing in Mongolian May influence the observed effects in a different manner. While the preview effect typically influences skipping rates, leading readers to be more likely to skip words that have been processed during preview (Vasilev et al., 2016), we did not observe a significant change in skipping rates under valid preview conditions. Despite this contrast, the overall trend of our results is consistent with the findings reported by Vasilev et al. (2016) (Bayesian meta-analysis estimated a preview benefit of 45 ms for GD, whereas our study observed a benefit of 40 ms). In summary, during vertical reading in Mongolian, readers can gain a preview benefit through parafoveal processing. However, this benefit primarily manifests as a reduction in the processing time for target words, rather than through increased word skipping. This phenomenon May be linked to the unique vertical writing system of Mongolian, particularly the mismatch between spatial word length and character count, as well as the seamless vertical connections between characters, which add complexity to parafoveal processing and May contribute to the reduced skipping rates observed. Further research is needed to explore how this vertical reading mode affects parafoveal information processing.

Another finding from our study is that the interaction between predictability and preview in Mongolian reading was not significant. Existing theories often debate whether the influence of contextual predictability is modulated by preview information, specifically whether the predictability effect relies on matching preview content (Parker et al., 2017; Staub and Goddard, 2019). Our results appear to support the view that the effect of contextual predictability is independent of preview (Parker et al., 2017). However, according to Parker et al. (2017), invalid preview can cause interference because it contradicts the reader’s expectations, leading to longer reading times and offsetting the benefits of contextual predictability. In contrast, our study found no significant interaction between contextual predictability and preview effects. Even under conditions of invalid preview, readers did not experience delays in reading time due to mismatched preview information, nor did it diminish the predictability effect. Regardless of whether the preview was valid or invalid, in Mongolian reading, the fixation times on target words were consistently faster in high predictability compared to low predictability.

We can further explain these findings using eye movement control models. According to the E-Z Reader model, word recognition occurs in two stages: familiarity check and lexical access. Word predictability affects both stages of this process. Compared to low-predictability words, high-predictability words are more easily anticipated and activated to a greater extent, thereby rapidly influencing the early stages of lexical processing. In this study, the features of Mongolian script and its reading direction not only made it harder to extract parafoveal information but also limited the ability to process parafoveal words, resulting in slower processing. Despite this, predictability still played a role in the early stages of Mongolian reading, showing predictability effects, although parafoveal processing was delayed. Moreover, the low skipping rate also shows the slow parafoveal processing in Mongolian reading. According to the E-Z Reader model, higher word predictability leads to a higher chance of the word being skipped and shorter fixation times. The parafoveal preview effect is characterized by shorter fixation times and higher skipping rates for words. However, in our study, skip rates were low regardless of whether the parafoveal preview was valid or not, and whether the context was highly predictable or not. Compared to invalid preview conditions, the parafoveal preview effect in Mongolian reading did not result in an increased skipping of target words, even when the parafoveal preview was valid and the target word received some degree of processing. Under both valid and invalid preview conditions, readers maintained a “reduced skipping” reading strategy in both high-predictability and low-predictability contexts. Therefore, this study did not find an interaction between predictability and preview in any time measures or skipping rates. This result does not definitively support or refute either perspective, but it does underscore that the interaction between predictability and parafoveal processing varies inconsistently across different writing systems. Therefore, discussions on the relationship between contextual predictability and preview need to acknowledge the specificity of different languages and their impact on research outcomes.

This study provides the first exploration of Mongolian language reading characteristics. It reveals that the reading process in Mongolian is influenced by its unique writing and reading styles. Compared to horizontally written alphabetic scripts, Mongolian exhibits distinctive features in parafoveal information processing, with lower word skipping rates. Additionally, the study investigates derived words in Mongolian as target stimuli. These words are typically formed by connecting root words with affixes, where the root carries most of the semantic information while the affixes provide additional grammatical information. The morphological structure of Mongolian derived words exhibits a certain complexity, including the positional information of morphemic units and the tightly connected word formation pattern, as well as the central semantic role of the root within the entire lexicon. Further investigation is needed to validate the impact of these features on the reading process. Subsequent studies could further explore predictive features in Mongolian reading by examining additional types of preview conditions.

## Data Availability

The original contributions presented in the study are included in the article/supplementary material, further inquiries can be directed to the corresponding author.
